# Neutrophils in COVID-19: Not Innocent Bystanders

**DOI:** 10.3389/fimmu.2022.864387

**Published:** 2022-06-01

**Authors:** Ellen McKenna, Richard Wubben, Johana M. Isaza-Correa, Ashanty M. Melo, Aisling Ui Mhaonaigh, Niall Conlon, James S. O’Donnell, Clíona Ní Cheallaigh, Tim Hurley, Nigel J. Stevenson, Mark A. Little, Eleanor J. Molloy

**Affiliations:** ^1^ Discipline of Paediatrics, Dublin Trinity College, The University of Dublin, Dublin, Ireland; ^2^ Paediatric Research Laboratory, Trinity Translational Medicine Institute (TTMI), St James’ Hospital, Dublin, Ireland; ^3^ Viral Immunology Group, School of Biochemistry and Immunology, Trinity Biomedical Sciences Institute, Dublin, Ireland; ^4^ Trinity Health Kidney Centre, Trinity Translational Medicine Institute (TTMI), Trinity College Dublin, Dublin, Ireland; ^5^ Department of Immunology, St James’ Hospital, Trinity College Dublin, Dublin, Ireland; ^6^ Irish Centre for Vascular Biology, Dublin, Ireland; ^7^ Department of Clinical Medicine, Trinity Centre for Health Science, Trinity College Dublin, Dublin, Ireland; ^8^ Department of Infectious Diseases, St James’s Hospital, Dublin, Ireland; ^9^ Neonatology, Coombe Women and Infant’s University Hospital, Dublin, Ireland; ^10^ National Children’s Research Centre, Children’s Hospital Ireland (CHI) at Crumlin, Dublin, Ireland; ^11^ Viral Immunology Group, Royal College of Surgeons in Ireland - Medical College of Bahrain, Al Muharraq, Bahrain; ^12^ Neonatology, Children’s Hospital Ireland (CHI) at Crumlin, Dublin, Ireland; ^13^ Paediatrics, Children’s Hospital Ireland (CHI) at Tallaght, Tallaght University Hospital, Dublin, Ireland

**Keywords:** neutrophil, COVID-19, SARS-CoV-2, innate immunity, inflammation

## Abstract

Unusually for a viral infection, the immunological phenotype of severe COVID-19 is characterised by a depleted lymphocyte and elevated neutrophil count, with the neutrophil-to-lymphocyte ratio correlating with disease severity. Neutrophils are the most abundant immune cell in the bloodstream and comprise different subpopulations with pleiotropic actions that are vital for host immunity. Unique neutrophil subpopulations vary in their capacity to mount antimicrobial responses, including NETosis (the generation of neutrophil extracellular traps), degranulation and *de novo* production of cytokines and chemokines. These processes play a role in antiviral immunity, but may also contribute to the local and systemic tissue damage seen in acute SARS-CoV-2 infection. Neutrophils also contribute to complications of COVID-19 such as thrombosis, acute respiratory distress syndrome and multisystem inflammatory disease in children. In this Progress review, we discuss the anti-viral and pathological roles of neutrophils in SARS-CoV-2 infection, and potential therapeutic strategies for COVID-19 that target neutrophil-mediated inflammatory responses.

## Introduction

Neutrophils are the first responders to infection and extravasate rapidly from the blood vessels into tissue. They are the most abundant leukocyte in blood, with about 10^11^ neutrophils produced by the bone marrow each day, representing 40-60% of circulating immune cells in healthy adults (1). Neutrophils kill pathogens using oxidative burst, degranulation, phagocytosis and the release of neutrophil extracellular traps (NETs) (2, 3). Their role is most prominent in bacterial infection but they can also contribute to antiviral immunity.

Severe disease in COVID-19 is associated to increased neutrophil-to-lymphocyte ratio and high expression of neutrophil-related cytokines IL-8 and IL-6 in serum, and neutrophilia has been described as a predictor of poor outcome (4–14). Peripheral blood neutrophil counts in patients with COVID-19, although not as elevated as bacterial pneumonia, are higher in severe COVID-19 compared with mild cases and most other viral infections (4, 15). Neutrophils are associated with the development of thrombosis and pulmonary infiltrates found in post-mortem samples following severe acute respiratory syndrome coronavirus 2 (SARS-CoV-2) (16–18). In this Progress review, we focus on emerging data on the roles of neutrophils in the pathogenesis and response to SARS-CoV-2.

## Neutrophils in COVID-19

An altered neutrophil-to-lymphocyte ratio occurs in many conditions such as cancer, cardiovascular disease, sepsis and inflammatory disorders, including Systemic lupus erythematosus (SLE) and psoriasis (19). Patients with COVID-19 with severe disease had significantly higher absolute neutrophil counts (8) similar to the neutrophilia in both Severe Acute Respiratory Syndrome (SARS) and Middle East Respiratory Syndrome (MERS) (20). The limited antiviral response in COVID-19 may exacerbate neutrophil infiltration, resulting in exuberant inflammation (21).

A small gene ontology (GO) analysis of COVID-19 infected cells indicated that neutrophil activation and degranulation are the most activated cellular immune processes in COVID-19, but did not play a role in the antibody-mediated elimination of SARS-CoV-2 in a passive immunisation model (22). Neutrophils contribute to hypersensitivity pneumonitis in SARS-CoV-2 infection and altered neutrophil immunometabolism, with accumulation of succinate correlating with disease severity (21). A rat coronavirus (RCoV) model demonstrated that neutrophils produce cytokines and chemokines in response to alveolar epithelial cell infection with SARS-CoV-2, resulting in an inflammatory response which contributes to lung injury (23).

## Neutrophil Extracellular Traps

Neutrophil extracellular traps (NETs) are web-like chromatin structures released by neutrophils to degrade virulence factors and kill bacteria. Once unregulated in sepsis or severe COVID-19, they induce multiple organ damage, including arterial hypotension, hypoxemia, coagulopathy, renal, neurological, and hepatic dysfunction as consequence of a NETs-associated cytokine storm (24–26). Silva et al. found that gasdermin inhibition with disulfiram or genic deletion decreases NETs formation with reduced multiple organ dysfunction and mortality in a sepsis model (27). NETs concentration was markedly increased in the tracheal aspirate and plasma of patients hospitalised with COVID-19 as well as in SARS-CoV-2-infected lung airways and alveoli, with spontaneous NETs production from their neutrophils (13, 28–32). SARS-CoV-2 can directly induce healthy neutrophils to release NETs *in vitro*, which increase pulmonary epithelium cell death (28). NETs also appear to drive neuroinflammation in Ischemic Brain Damage (IBD) and IBD following COVID-19, by affecting the blood-brain barrier, promoting thrombosis, and by inducing neuronal damage through extruded NETs components, NETs-IL-1 loop and IL-17 cascades (33, 34), making them a promising target for therapy.

**Figure 1:**
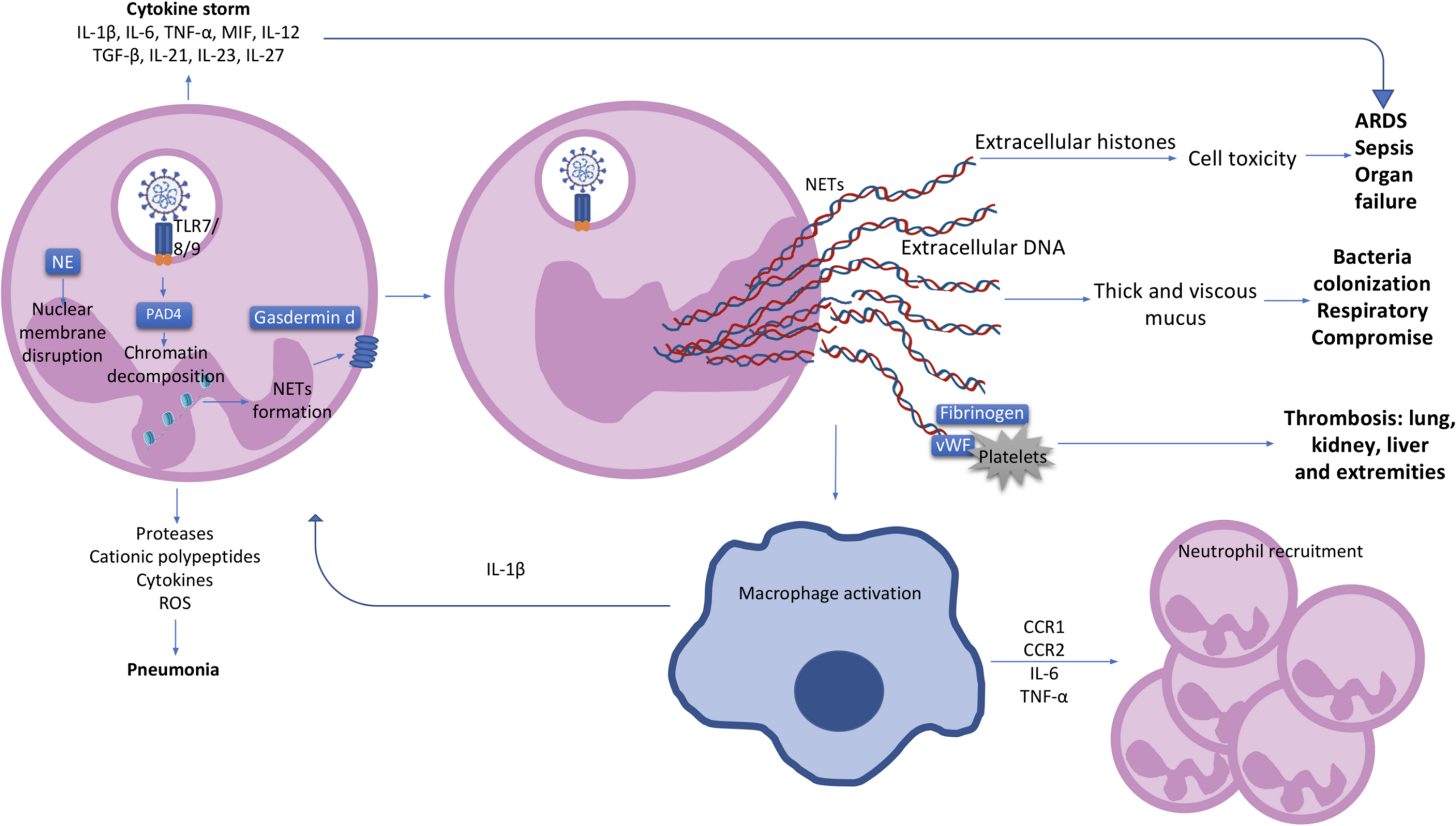
The neutrophil and clinical characteristics of COVID-19 patients. Activated neutrophils can produce cytokines such as IL-1β, IL-6, TNF-α, MIF, IL-12, TGF-β, IL-21, IL-23 and IL-27, contributing to a cytokine storm and the further development of ARDS and organ failure in COVID-19 patients. Pneumonia in COVID-19 patients is most likely to be caused by the production of proteases, cationic polypeptides, cytokines and ROS by neutrophils. Upon SARS-CoV-2 recognition by TLR7/8/9, protein arginase deiminase 4 (PAD4) is activated, which induces chromatin decondensation through histones citrullination and consequently NETs formation. Neutrophil nuclear membrane is disrupted by neutrophil elastase (NE) and gasdermin D which facilitates the formation of a pore in the neutrophil cell membrane and mediates release of the contents of NETs into the extracellular space. NETs induce macrophage activation and IL-1β production resulting in a positive loop with neutrophils and NETs formation. Macrophages also secrete CCR1, CCR2, IL-6 and TNF-α leading to further neutrophil recruitment. Extracellular histones presented in NETs causes cell cytotoxicity contributing with ARDS, sepsis and organ failure observed in COVID-19 patients. Extracellular DNA induces thick and viscous mucus production allowing bacteria colonization and respiratory failure. NETs also interact with fibrinogen, VWF and platelets causing thrombosis in several organs such as lung, kidney, liver and extremities. IL, interleukin; TNF, tumour necrosis factor; MIF, macrophage migration inhibitory factor; ARDS, acute respiratory distress syndrome; ROS, reactive oxygen species; PAD4, protein arginase deiminase 4; NETs, neutrophil extracellular trap; NE, neutrophil elastase; CCR, chemokine receptor; DNA, deoxyribonucleic acid; VWF, von Willebrand factor.

The first step in NETosis is cellular activation *via* pattern recognition receptors (PRR) such as Toll-like receptors 4 (TLR4), TLR7 and TLR8 in viral infections (24, 35, 36). Reactive oxygen species (ROS) are subsequently produced, resulting in the activation of protein arginase deiminase 4 (PAD4) which is responsible for chromatin decondensation (24, 37). Neutrophil elastase (NE), a granule protein, induces neutrophil nuclear membrane break down while granule protein gasdermin D facilitates pore formation in the cell membrane and mediates release of NETs into the extracellular space (**Figure 1**) (24, 31). NETs do play a role in viral clearance, but excessive NETs production exacerbates inflammation in acute respiratory distress syndrome (ARDS) and contributes to microvascular thrombosis (**Figure 1**) (38). These is potentially related to over-activation of the Stimulator of interferon genes (STING) pathway through cyclic GMP-AMP synthase (cGAS) in phagosomes, and by SARS-CoV-2 infection itself through Angiotensin-Converting Enzyme 2 (ACE2)-angiotensin II (39, 40). Pharmacological activation of the STING pathway may also regulate the effects of SARS-CoV-2 infection (41). NETs can also have different proteins cargo associated to their deoxyribonucleic acid (DNA), citrullinated histone 3 (cit-H3), NE, and myeloperoxidase (MPO) structure which can influence the type of immune response triggered (42). Severe COVID-19 patients were shown to have higher expression of the alarmin nuclear protein High mobility group box 1 (HMGB1), antiviral molecules like ISG-15 and LL-37, or functionally active tissue factor (TF) as protein cargo in NETs, produced mostly by normal density granulocytes (NDG) (43, 44). These cargo molecules induced thrombogenic activity and differential cytokines expression (43, 44).

## Inflammasome Activation in COVID-19

**Figure 2:**
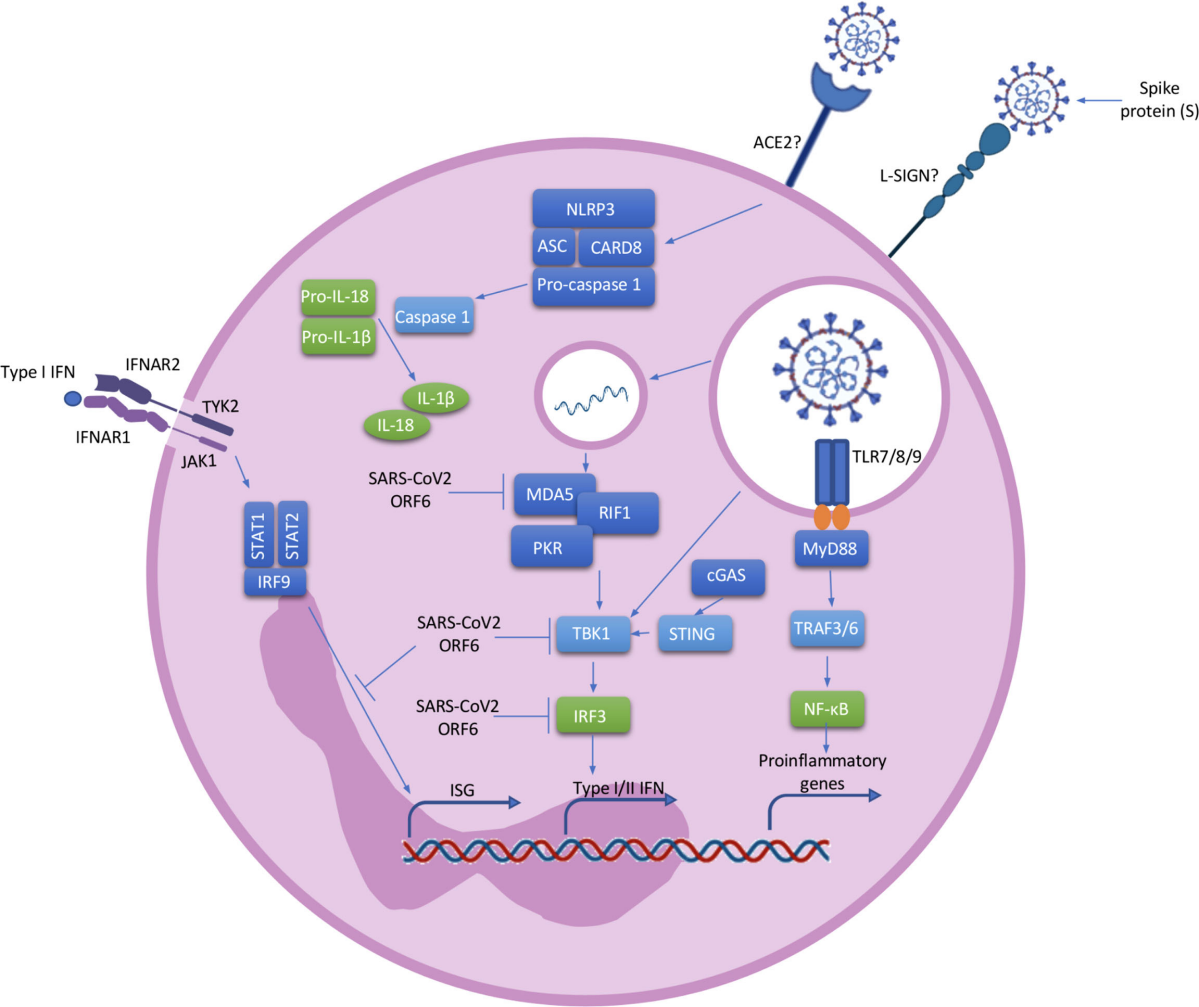
Neutrophil activation. ACE2 or L-SING receptors on neutrophils most likely recognise SARS-CoV2 *via* a spike (S) protein on its surface. Once the virus enters the cell, ssRNA viruses such as SARS-CoV-2 are recognised by TLR 7/8/9 which induce the activation of the MyD88 pathway. MyD88 activates TRAF3 and TRAF6 which result in the transcription of NF-κB and IRF7 associated genes. The activated NF-κB pathway leads to the transcriptional induction of proinflammatory cytokines, chemokines and additional inflammatory mediators in neutrophils. In addition, cytosolic viral RNA recruiting MDA5, RIF1 and PKR lead to the activation of TBK1 and the further activation of IRF3 resulting in the transcription of type I/II IFN genes. The positive stimulatory loop by type I IFN induces the production of more IFNs through the JAK/STAT pathway and the induction of Interferon Stimulated Genes (ISG). At the same time, SARS-CoV-2 possess ORF6, an accessory protein antagonist of IFNs by the inhibition of MDA5, TBK1, IRF3 and IRF9. ssRNA viruses also cause the recruitment of the NLRP3 inflammasome complex and the further activation of pro-caspase-1 resulting in the cleavage of pro-IL-1β and pro-IL-18 into the active forms. ACE2, angiotensin-Converting Enzyme 2; L-SING, L-Specific Intercellular adhesion molecule-3-Grabbing Non-integrin/CD209L; RNA, ribonucleic acid; ssRNA, Single-stranded RNA; TLR, toll-like receptor; MyD88, myeloid differentiation primary response 88; TRAF, tumor necrosis factor receptor (TNF-R)-associated factor; NF-κB, nuclear factor kappa B; IRF, Interferon Regulatory Factor; MDA, melanoma differentiation-associated protein; RIF, Replication Timing Regulatory Factor; PKR, protein kinase R; TBK, TANK Binding Kinase; IFN, interferon; ISG, Interferon Stimulated Genes; JAK-STAT, janus kinase; ORF, open Reading Frame; NLRP3, nod like receptor family, pyrin domain containing 3; IL, interleukin.

COVID-19 is characterised by a cytokine storm and the Pyrin domain containing 3 (NLRP3) inflammasome has been implicated. The inflammasomes are molecular mechanism involving multiprotein complexes which regulate the production of pro-inflammatory cytokines. NLRP3, a member of the nucleotide oligomerization domain (NOD)-like receptor (NLR) family, is present in neutrophils (17). After NLRP3 activation, pro-caspase 1 is cleaved to the active form caspase 1, leading to the cleavage of pro-inflammatory pro-IL-1β and pro-IL-18 into the active forms (**Figure 2**) (45). Single-stranded ribonucleic acid (ssRNA) viruses, such as SARS-CoV-2, induce Nuclear factor kappa B (NF-κB) activation and the further production of pro-IL-1β and pro-IL-18 (45, 46). Simultaneously, ROS and Adenosine 5’-triphosphate (ATP) produced by mitochondria trigger NLRP3 inflammasome assembly (46). Active NLRP3 inflammasome is present in peripheral blood mononuclear cells (PBMCs) and post-mortem tissues of COVID-19 patients, and high expression of its derived products such as Casp1p20 and IL-18 were seen to correlate with disease severity and poor clinical outcome (47). NLRP3 inflammasome activation has also been described in neutrophils of severe COVID-19 patients (48). Aymonnier et al. found that neutrophils from COVID-19 patients with respiratory failure demonstrated NLRP3 inflammasome molecule Apoptosis-associated speck-like protein containing a CARD (ASC) specks, and their early formation in NETosis. In patients with severe COVID-19 neutrophils with intact multilobulated nuclei, ASC specks formation and histone H3 citrullination was elevated (48). In a murine model they also showed transient presence of ASC specks at the microtubule organizing center, before nuclear rounding, early in NETosis (48). In addition, SARS-CoV-2 has been shown to directly activate the NLRP3 inflammasome through viroporin protein 3a, which most likely acts by the formation of K^+^ and Ca^+^ channels (49). Such direct activation of the inflammasome leads to the production of IL-1β and IL-18, perpetuating inflammation and resulting in further neutrophil activation (50). NLRP3 inflammasome activation in the blood of patients reveals an impaired immature neutrophil response in severe COVID-19. Inflammasome signature analysis in circulating myeloid cells allows COVID-19 patients to be stratified and predicts evolution of disease severity (51).

## Neutrophil Subsets in COVID-19

Heterogeneity within the neutrophil population during infection has been demonstrated in multiple diseases, and different subsets have defined roles in influencing the inflammatory response (38, 52). Neutrophil subsets varying in their density, maturity and expression of surface markers have been reported in COVID-19 (53, 54). Classically, in sepsis, immature neutrophils are released from the bone marrow and Carissimo et al. found increased immature neutrophils in whole blood that correlated with increased IL-6 and IP-10, and COVID-19 disease severity (55). The ratio of immature neutrophils to gamma delta (Vδ)2 T cells could predict severe COVID-19 (55). Additionally, a shift toward immature neutrophils as the driver of hyperinflammation is associated with severe COVID-19 disease (56).

Recently there has been a renewed interest in immunomodulatory neutrophil subsets, specifically in the field of cancer, SLE and sepsis, including low density granulocytes (LDGs) and myeloid derived suppressor cells (MDSCs) (38), but there is not a consensus on nomenclature and classification (57). MDSCs are a mixed population of mature and immature cells with differing immunomodulatory roles (58). There is a lack of clarity on the phenotypical and functional characteristics of MDSCs and their relationship to LDGs but their defining characteristic is suppression of the adaptive immune response (59). MDSC expansion is linked to G-CSF, a cytokine increased in the lungs of COVID-19 patients (60) and almost 90% of mononuclear cells in the severe disease cohort were MDSCs. Proportion of LDGs increases with disease severity in COVID-19 patients, as well as their production of NETs when compared to healthy controls (43, 61).

Morrisey et al. described a population of LDGs correlating with disease severity and hypercoagulable state in COVID-19 patients (53). A population of CD45^+^CD66b^+^CD16^Int^CD44^low^CD11b^Int^ LDGs was found in patients with severe disease, which displayed enhanced phagocytic capacity, spontaneous NETs formation and elevated cytokine production. Similarly, an immune-suppressive CD16^bright^/CD62L^dim^ neutrophil subtype was increased in patients developing pulmonary embolism (PE) on the day of ICU admission (54). Using whole blood transcriptomics analysis, increased NLRP3 inflammasome, monocytes and LDGs were found in the lungs of COVID-19 patients, and neutrophil activation-associated signatures correlated to disease severity (62). In COVID-19, immature neutrophils are expanded and show increased programmed death ligand (PD-L) 1, which suppresses T cells, and reduced oxidative burst functions with no change in phagocytosis in severe COVID-19 (63). Chevrier et al. found higher LDGs were present in COVID-19 patients early in the course of the disease and decreased in convalescence using mass cytometry and serum proteomics, but CD16^low^ neutrophil population remained expanded over the disease course (64). COVID-19 induced-ARDS is associated with MDSC expansion, reduced lymphocyte function and arginine shortage, through increased arginase activity, therefore arginase supplementation may be therapeutic (65). Further study into the role of neutrophil subsets in COVID-19 is warranted, potentially as biomarkers of disease severity, or as new targets for therapeutic approaches.

## Neutrophil Response to SARS-CoV-2

### Does SARS-CoV-2 Actively Infect Neutrophils?

Although neutrophils express the L-SIGN and DC-SIGN C-type lectins receptors that have been suggested to act as entry receptors for SARS-CoV-2, there is conflicting evidence about active infection of neutrophils with the virus. In other ssRNA viruses such as West Nile and influenza virus neutrophils serve as an important viral reservoir and contain actively replicating virus, and studies with human immunodeficiency virus (HIV) and Respiratory syncytial virus (RSV) viral models suggest that neutrophils can internalise virus without productive infection (66). Neutrophils are important for viral detection and initiation of downstream effector immune pathways but the replicative ability of ssRNA virus SARS-CoV-2 within neutrophils is not known.

ACE2 is the primary cell entry receptor for SARS-CoV-2 and ACE2 deficiency is associated with worse outcomes in COVID-19 (67). Entry of SARS-CoV2 into the cells following membrane fusion majorly down-regulates ACE2 receptors, with loss of the catalytic effect of these receptors at the external site of the membrane (68). This induces increased pulmonary inflammation and coagulation due to enhanced and unopposed angiotensin II effects. ACE2 down-regulation induced by viral invasion may be especially detrimental in people with baseline ACE2 deficiency (68). Following viral entry, the additional ACE2 deficiency may exacerbate the dysregulation between ACE→Angiotensin II→AT1 receptor axis (potentially adverse) and the ACE2→Angiotensin→Mas receptor axis (negative regulator of angiotensin II activity, potentially protective recombinant ACE2) (68). Therefore, angiotensin and angiotensin II type 1 receptor blockers may be beneficial in patients with severe SARS-CoV-2 (68). However, two large cohort studies showed that angiotensin-converting enzyme inhibitors (ACEIs)/angiotensin receptor blockers (ARBs) use was not associated with increased SARS-CoV-2 infection, but was in fact associated with a lower risk of all-cause mortality in hospitalized patients (69, 70). Further studies are needed to test the protective effects of ACEIs/ARBs in COVID-19 (69, 70). NETs triggered by SARS-CoV-2 depend on ACE2, serine protease TMPRSS2, virus replication, and PAD-4 (28). ACE is important in neutrophil antibacterial activity. Veras at al found that NETosis was facilitated in neutrophils in patients with COVID-19 (28). Neutrophils express ACE2 similar to other immune cells and it is postulated that allows the virus-triggered cell activation and NETosis (28). Knockout of this gene in mice or treatment with an ACE inhibitor increased susceptible to bacterial infection by methicillin-resistant *Staphylococcus aureus* (MRSA). Mice overexpressing ACE in neutrophils have increased killing of MRSA *Pseudomonas aeruginosa*, and *Klebsiella pneumoniae*, with increased neutrophil production of reactive oxygen species (ROS) independent of the angiotensin II AT1 receptor (71).

## Dysfunctional Neutrophil Activation in COVID-19

Neutrophils express all known Toll-like receptors (TLRs) with the exception of TLR3 (72). TLR7, TLR8 and TLR9 are involved in the detection of ssRNA viruses such as SARS-COV-2 (73). Activation of these receptors leads to downstream activation of NF-κB and interferon regulatory factor (IRF7), and the subsequent production of pro-inflammatory cytokines and chemokines in neutrophils (**Figure 2**) (74). In conjunction with neutrophils, these pro-inflammatory cytokines and chemokines drive the characteristic hyperinflammation and pulmonary infiltration seen in severe COVID-19 (74). Neutrophils also produce type 1 interferons (IFN-α/IFNβ) through the activation of IRF proteins (75) and this broad, but dysregulated, pro-inflammatory and antiviral response puts selective pressure on these highly pathogenic respiratory viruses. The host response to SARS-CoV-2 has also been broadly defined as a significantly depleted type 1 IFN response, with a consistent upregulation of chemotactic signals (CCL8, CCL2, CXCL2, CXCL8 and CXCL9), most of which are key mediators of neutrophil recruitment. Liao et al. found that in the lungs of patients with severe COVID-19, macrophages exacerbate inflammation by producing chemokines that recruit neutrophils to the site of infection through chemokine receptors CC-chemokine receptor 1 (CCR1) and C-X-C chemokine receptor type 2 (CXCR2) (57). Using a SARS-CoV-2 animal model early induction of CXCL9 and CCL8 was found consistent with observations in primary human bronchial epithelial cells infected with SARS-CoV-2. At day 7, despite waning levels of virus, elevated CCR5, CCL2, CXCL9 and IL-6 were found in the animal model, suggesting neutrophil-mediated inflammation may persist after the virus has been cleared (76). This may correlate with the clinical findings of persistent symptoms and fatigue with post-viral infection complications in some patients.

The loss of IFN signalling is vital to understanding why SARS-CoV-2 elicits such a potent inflammatory and neutrophilic chemotactic response. For instance, bats appear to limit the inflammatory and neutrophilic chemotactic response when infected with coronaviruses endemic in the bat population (77). Banerjee et al. have proposed that bats possess repressors of NF-κB signalling, a potent inductor of pro-inflammatory and chemotactic responses, allowing these strains of the viruses to become endemic in the population. However, unlike bats, humans lack this repressor activity rendering us susceptible to this uncontrollable neutrophil-mediated inflammatory response following viral infection (77).

## Neutrophils and Thrombosis

Coagulation cascade activation is a common finding in patients with COVID-19 and is associated with disease severity (78). Elevated levels of fibrin D-dimer degradation products, a marker of fibrin degradation indicating overactive coagulation, correlates with a worse clinical outcome (79). High plasma levels of plasminogen activator (tPA) and plasminogen activator inhibitor-1 (PAI-1) in hospitalised COVID-19 patients had strong correlations with neutrophil counts and activation, and extremely high levels of tPA increasing fibrinolysis (80). Plasmatic matrix metalloproteinase-9 (MMP-9) was likewise increased in COVID-19 patients which induced platelet and neutrophil activation, and NETs formation *in vitro* (81). Post-mortem studies have consistently shown that micro-thrombi are present throughout the pulmonary vasculature (82). Collectively, these data suggest that coagulation activation and vasculopathy within the lungs (pulmonary intravascular coagulopathy [PIC]) plays a role in modulating COVID-19 pathogenesis (78). The biological mechanisms through which SARS-CoV-2 infection causes PIC within the lung blood vessels remain poorly understood (83). However, recent autopsy studies have reported significant endothelial cell (EC) damage, apoptosis, loss of tight junctions and separation from the basement membrane (84). Local inflammation and dysregulated pro-inflammatory cytokine generation within the lungs are a major factor as well as local hypoxia and complement activation, which significantly enhance procoagulant pathways and downregulate anticoagulant pathways *in vivo*. Moreover, ECs express the ACE2 receptor through which SARS-CoV-2 gains entry into cells, and electron microscopy studies have reported viral inclusion bodies within ECs.

Neutrophils and platelets are key modulators of thrombosis. Significant NETosis is found in patients with severe COVID-19 and is important in thrombus aetiology (85). NETs can bind to platelets, triggering platelet activation, and through their citrullinated histone H3 (citH3) they can also interact with procoagulant von Willebrand factor (VWF) (85). In addition to their effects on primary hemostasis, NETs also enhance local thrombin generation. In particular, NETs initiate coagulation activation through the alternative contact pathway and trigger thrombin generation by enhancing the intrinsic tissue-factor dependent pathway. NETs have also been described to over-activate the STING pathway through the cGAS sensor in phagosomes (40). The over-activation of the STING-pathway increases hyper-coagulability *via* interferon-β and tissue factor, released by monocytes-macrophages, and can be inhibited upstream the STING-pathway by aspirin, intravenous immunoglobulins and Vitamin-D (40). NETs histones can activate platelets by stimulating platelet TLR4 and TLR2; neutrophils can bind to these active platelets through surface glycoprotein Ib to induce NETosis and, consequently, result in thrombosis (85). Platelet activation is associated with disease severity in COVID-19 (86). Finally, NETosis has potent pro-inflammatory effects on ECs, which serve to attenuate the normal ability of ECs to regulate procoagulant pathways (87, 88). NETs and thrombosis have been implicated in several disorders including cancer, SLE, rheumatoid arthritis (RA), atherosclerosis and ischemic stroke. NETs have been shown to invade microthrombi in septic patients and contribute to organ damage, hence it is likely that neutrophils are a mediator of organ dysfunction in COVID-19 (31).

## Neutrophils and COVID-19 in Children

The severity of COVID-19 differs between age-groups, and children, especially neonates, exhibit milder disease with only a small proportion require intensive care with acute respiratory illness. There are many theories about this discrepancy, which is also seen with other similar viral illnesses, and the decreased expression of ACE2 and NETs formation may be contributory (89). However, a multisystem inflammatory disease in children (MIS-C) or paediatric multisystem inflammatory syndrome temporally associated with COVID-19 (PIMS-TS) has emerged in children, occurring weeks after the primary infection with SARS-Cov-2, that can lead to serious and life-threatening illness in previously healthy children (90). There is no internationally accepted single definition of MIS-C/PIMS-TS, but most case definitions require multi-organ dysfunction, systemic inflammation evidence of recent a SARS-CoV-2 infection, and the exclusion of other causes. The clinical presentation and laboratory findings in MIS-C are similar to Kawasaki’s disease and toxic shock syndrome, and considered to be a spectrum of disease (90).

Similar to adults with COVID-19, neutrophilia and lymphocytopenia are common in MIS-C. Neutrophils play a key functional role in Kawasaki disease with recent descriptions of NETosis and neutrophil activation in the form of CD11b and CD64 production (91). Neutrophil counts predict responsiveness of patients with Kawasaki disease to intravenous immunoglobulin therapy, also used in MIS-C (92, 93). Neutrophils activation marker Fc γ receptor I (FcγRI; CD64) was described to be highly expressed on neutrophils of treatment-naive MIS-C patients in acute phase compared with healthy controls (94). These patients also showed increase levels of the neutrophils chemoattractant cytokine IL-8 (94). Ramaswamy et al. talk of a potential myeloid dysfunction in MIS-C patients based on the high expression of alarmin-related S100A genes in neutrophils and monocytes, and the significant reduction in key antigen-presentation molecules such as HLA class II and CD86 (95). Additional research is required to fully understand the role of neutrophils in MIS-C and to determine whether treatments used in Kawasaki disease such as intravenous immunoglobulin therapy could also be used with MIS-C patients.

## Therapeutic Targeting of Neutrophils

### Targeting Cytokines

The efficacy of targeting cytokines produced by various immune cells, including neutrophils, is being explored in ongoing clinical trials. Neutrophils produce IL-6, and IL-6 inhibitor tocilizumab has been shown to decreases neutrophil survival and lipopolysaccharides (LPS)-induced oxidative burst, as well as neutrophil release from the bone marrow and lung demargination (96, 97). Tocilizumab has been approved by the United States Food and Drug Administration (FDA) for use in COVID-19 patients and decreased mortality, poor outcome and mechanical ventilation (98, 99). Clazakizumab also targets IL-6 and is currently being evaluated for safety in several clinical trials of patients with life-threatening COVID-19 (**Table 1**). The interleukin-6 receptor inhibitors (IL6ri) sarilumab or tocilizumab decreased intubation and mortality in a study including 255 patients with COVID-19 (100). Doxycycline (a tetracycline) reduces IL-6, IL-1β and TNF-α levels, however, doxycycline treatment did not have a significant clinical impact on time to recovery, hospital admissions or deaths related to COVID-19 in patients with high risk to adverse outcomes (101).

**Table 1:** Clinical trials therapeutically targeting neutrophils.

Therapeutic target	Type of drug	Drug name	Effect on Neutrophils	Reference number
IL-6	Anti-IL-6	Clazakimumab	Reduces inflammation produced by neutrophils and other immune cells	NCT04363502 NCT04381052 NCT04343989
	Anti-IL-6	Tocilizumab		NCT04403685
	Anti-IL-6	Siltuximab		NCT04329650
	Anti-IL-6	Olokizumab		NCT04452474
	Anti-IL-6R	Sarilumab		NCT04357860
GM-CSF	Monoclonal antibody-anti-GM-CSF	Lenzilumab	Blocks neutrophils recruitment	NCT04351152
	Monoclonal antibody-anti-GM-CSF	Mavrilimumab		NCT04397497
	Monoclonal antibody-anti-GM-CSF	TJ003234		NCT04341116
	Monoclonal antibody-anti-GM-CSF	Gimsilumab		NCT04351243
	GM-CSF	Sargramostim	Recruits neutrophils	NCT04400929 NCT04411680 NCT04326920 NCT04400929
NLRP3 inflammasome	Inhibitor of NLRP3 inflammasome	Colchicine	Reduces NLRP3 inflammasome activated by neutrophils	NCT04322682 NCT04350320 NCT04322565 NCT04326790 NCT04367168 NCT04381936
	Inhibitor of NLRP3 inflammasome	Tranilast	Reduces hyperinflammation and organ damage	ChiCTR2000030002
NLRP3 inflammasome	Inhibitor of NLRP3 inflammasome	Dapansutrile	Reduces hyperinflammation and organ damage	NCT04540120
IL-1β	Anti-IL-1β monoclonal antibody	Canakinumab	Reduces hyperinflammation and organ damage	NCT04365153 NCT04348448 NCT04362813
IL-1	IL-1 receptor antagonist	Anakinra	Reduces hyperinflammation and organ damage	NCT04339712 NCT04324021 NCT04341584
IFN-y	Anti-IFN-y	Emapalumab	Inhibits activation of neutrophils	NCT04324021
TLR4	TLR4 inhibitor	EB05	Reduces hyperinflammation and organ damage	NCT04401475
NETs	rhDNase1	Dornase alfa	Promotes clearance of NETs	NCT04432987 NCT04359654 NCT04355364 NCT04409925 NCT04402970 NCT04402944
	NE inhibitor	13 cis retinoic acid	Promotes clearance of NETs	NCT04396067
	NE inhibitor	Alvelestat		NCT04539795
	NE inhibitor	Brensocatib		NCT04817332
JAK-STAT	JAK1/2 inhibitor	Ruxolitinib	Reduces inflammation produced by neutrophils and other immune cells.	NCT04334044 NCT04348071 NCT04355793 NCT04366232 NCT04362137
JAK-STAT	JAK1/2 inhibitor	Baricitinib Tofacitinib	Reduces inflammation produced by neutrophils and other immune cells.	NCT04320277 NCT04340232 NCT04321993 NCT04401579 NCT04469114 NCT04750317
Angiotensin receptor	Angiotensin receptor blocker	Telmisartan	Reduces oxidative stress. Inhibits NADPH oxidase in neutrophils.	NCT04360551 NCT04355936
	Angiotensin II receptor antagonist	Losartan	Blocks neutrophils recruitment	NCT04340557 NCT04328012
	Angiotensin II receptor antagonist	Valsartan	Reduces oxidative stress. Inhibits NADPH oxidase in neutrophils.	NCT04335786
	Inhibitor of the spike protein serine proteases	Alpha-1 antitrypsin	Blocks neutrophils recruitment	NCT04385836
Neutrophil	Calcium-release activated calcium (CRAC) channel inhibitor	CM4620-IE	Blocks neutrophils recruitment	NCT04345614
	Neutrophil viability modulator	Intravenous immunoglobulin (IVIG)	Neutrophil viability modulator	NCT04432324 NCT04411667 NCT04383548 NCT04403269
		L-MOD	Neutrophil viability modulator	NCT04353674
	Neutrophil chemotaxis inhibitor	Lenalidomide Dexamethasone	Blocks neutrophils recruitment	NCT04361643 NCT04325061 NCT04395105 NCT04360876 NCT04344730
IL-6, IL-8, IL-1β and TNF-α	Modulates IL-8, TNF-α, IL-1β and IL-6 gene expression	Doxycycline	Reduces inflammation produced by neutrophils and other immune cells	NCT04371952
IL-17A	Binds interleukin 17A and neutralizes it	Ixekizumab	Reduces inflammation produced by neutrophils and other immune cells	NCT04724629
Anti-inflammatory and anti-fibrotic agent	Monoclonal antibody	TB006	Reduces inflammation produced by neutrophils and other immune cells	NCT04801056

IL, interleukin; GM-CSF, granulocyte-macrophage colony-stimulating factor; NLRP3, nod like receptor family, pyrin domain containing 3; IFN, interferon; TLR, toll-like receptor; NET, neutrophil extracellular trap; JAK-STAT, janus kinase; TNF, tumour necrosis factor.

Granulocyte-macrophage colony-stimulating factor (GM-CSF) is involved in neutrophil recruitment, survival, IL-6 release and priming for NETosis (102, 103). Mavrilimumab, an anti-GM-CSF receptor-α monoclonal antibody, improved clinical outcomes in patients with COVID-19 pneumonia and systemic hyperinflammation (104). In contrast, sargramostim, a recombinant human GM-CSF is under investigation, to improve the immune response by recruiting neutrophils, dendritic cells and macrophages to fight the virus and to repair tissue damage (**Table 1**), although there may be significant risks including neurotoxicity (103). GM-CSF also induces the expansion of immunosuppressive MDSCs, which impair NK cells, CD8+ T cells and increase proliferation of immunosuppressive T regulatory (Treg) cells (105, 106). GM-CSF stimulates the expression of IL-1β, IL-6, TNFα and other pro-inflammatory cytokines and chemokines, therefore, its inhibition would more broadly dampen hyperinflammation than therapy for IL-6 alone. In patients with rheumatoid arthritis this strategy is used for those unresponsive to anti-TNF therapy or tocilizumab (106). Cytokine signalling pathways are targeted by using inhibitors of JAK1/JAK2, to potentially reduce inflammation (**Table 1**). Clinical trials using JAK1/JAKK2 inhibitor Baricitinib showed reduction in 30-day mortality in over 70s with moderate-to-severe COVID-19 pneumonia, and combined with Remdesivir decreased recovery time and reduced 28-day mortality, serious events and new infections (107, 108). Reduction in the risk of death or respiratory failure was also described in a clinical trial including 289 COVID-19 patients when comparing the effects of JAK inhibitor Tofacitinib with a placebo (109).

NLRP3 inflammasome activation in neutrophils is implicated with pulmonary inflammation and inhibition with MCC950 inhibited IL-1β in the lungs of cystic fibrosis mice (110). Tranilast is the first NLRP3 inflammasome inhibitor in clinical trials in the Chinese Clinical Trial Registry. Interleukin-1 blockade with canakinumab treatment increases neutrophil apoptosis and decreases pro-inflammatory signalling in the IL-1β pathway using gene expression and pathway data (111). Canakinumab, is another FDA approved drug under investigation in clinical trials, and may help reduce respiratory and cardiac damage. Colchicine targets the neutrophil and monocyte NLRP3 inflammasome, hence attenuating activation of IL-1β (112). However, no significant differences were seen in primary (disease progression or mortality) or secondary (time to discharge, proportion of patients discharged, time in Intensive Care unit (ICU) or duration of hospitalisation) outcomes in two separated clinical trials comparing patients who were given colchicine to placebo/usual care treated patients (113, 114). Anakinra (commercially known as Kineret) is an FDA approved human IL-1RA (inflammasome-regulated immune response inhibitor of IL-1) which may reduce hyperinflammation and organ damage (**Table 1**) (112). Clinical trials using anakinra as treatment for COVID-19 have reported conflicting results. One study described lower risk of clinical progression in patients who received anakinra compared to placebo, while other study reports no effect of anakinra treatment on in-hospital mortality or days of organ support (115–117). However, the European Medicines Agency (EMA) recommended the use of anakinra in December 2021, specifically for COVID-19 adult patients at risk of developing severe respiratory failure or with pneumonia requiring supplemental oxygen (118).

### Intravenous Immunoglobulin (IVIG) and Corticosteroids

IVIG are purified IgG made from a pool of plasma from healthy donors (119) and modulate neutrophil viability through agonistic antibodies anti-Fas and Siglec-9 (120). It may also decrease neutrophil activation and NETs formation and mitigate vascular injury (121). IVIG has been tested in clinical trials in patients with COVID-19 (**Table 1**) and has shown to have therapeutic value (121). Similar positive effects of IVIG have been described in children with Kawasaki’s disease and MIS-C. However, ambiguity exists about dose dependent pro/anti-inflammatory effects as high dose IVIG is anti-inflammatory while a lower dose is considered pro-inflammatory (122). The widespread utility of this therapy may be precluded by plasma shortage, as it is also use as treatment in immunodeficiencies and inflammatory disorders. Treatment of healthy neutrophils with IVIG decreased NETosis and ROS production but enhanced phagocytosis (122).

The efficacy of treating COVID-19 patients with corticosteroids remains controversial. Lomas et al. have demonstrated that dexamethasone can inhibit neutrophil chemotaxis *in vitro* and *in vivo* (123). A variety of studies hypothesise that this anti-inflammatory drug may be effective in reducing ARDS and respiratory failure in COVID-19 patients (**Table 1**). The randomised evaluation of COVID-19 therapy (RECOVERY) trial in hospitalized COVID-19 patients found that treatment with dexamethasone results in a lower 28-day mortality for patients receiving oxygen only or ventilation, though no explanation of the mechanism for this was provided (124). Neutrophil-to-Lymphocyte ratio was reduced in patients treated with corticosteroids for COVID-19.

### Targeting NETs

The targeting of neutrophil extracellular traps with dornase alfa, a human recombinant deoxyribonuclease (DNAse) enzyme, degrades DNA and promotes the clearance of NETs and has been used in patients with cystic fibrosis (125). Several studies are investigating the use of dornase alfa to improve pulmonary function in severe COVID-19 with ARDS (**Table 1**) (125). Similarly, all-trans retinoic acid, an inhibitor of NE (granular component involved in NETosis), is also being explored to improve lung injury in COVID-19 patients. COVID-19 is associated with a significant neutrophil NETs burden and targeting NETs-driven IL-1 signalling, using the IL-1 receptor antagonist, decreased NETosis and may modulate inflammation.

## Conclusion

The clinical syndrome of severe COVID-19 has several unique features, including, unusually for a viral infection, an increased neutrophil-lymphocyte ratio. Neutrophils play a role in viral clearance in terms of NETs and the production of IFN. However, neutrophils can have detrimental effects by aiding the pathogenesis of SARS-CoV-2 and exacerbating complications of COVID-19 such as ARDS, thrombosis and MIS-C. Understanding the role of neutrophils in the pathogenesis of severe COVID-19 may lead to identification of key therapeutic targets and/or biomarkers for early identification of patients who may benefit from immunomodulatory agents to control hyperinflammation and reduce mortality rates.

## Author Contributions

EM and AMM have performed literature research, designed the review layout, wrote, and revised the review. RW, AUM, NC, JO’D, CNC, TH, and NS have performed literature research, wrote, and revised the review. JI-C designed the review layout, and revised the review. ML has performed literature research, designed the review layout, wrote, and revised the review. EJM has performed literature research, designed the review layout, wrote, and revised the review. All authors agree to be accountable for the content of the work. All authors contributed to the article and approved the submitted version.

## Conflict of Interest

The authors declare that the research was conducted in the absence of any commercial or financial relationships that could be construed as a potential conflict of interest.

## Publisher’s Note

All claims expressed in this article are solely those of the authors and do not necessarily represent those of their affiliated organizations, or those of the publisher, the editors and the reviewers. Any product that may be evaluated in this article, or claim that may be made by its manufacturer, is not guaranteed or endorsed by the publisher.
